# Extraction of Bridge Fundamental Frequencies Utilizing a Smartphone MEMS Accelerometer

**DOI:** 10.3390/s19143143

**Published:** 2019-07-17

**Authors:** Ahmed Elhattab, Nasim Uddin, Eugene OBrien

**Affiliations:** 1 Department of Civil, Construction, and Environmental Engineering, The University of Alabama at Birmingham, 1075 13th St S, Birmingham, AL 35205, USA; 2 School of Civil Engineering, University College Dublin, Newstead Block B, Belfield, Dublin D04V1W8, Ireland

**Keywords:** stochastic resonance, bridge inspection, structural health monitoring, SHM, bridge health monitoring, frequency independent stochastic resonance, SHM using smartphones

## Abstract

Smartphone MEMS (Micro Electrical Mechanical System) accelerometers have relatively low sensitivity and high output noise density. Therefore, it cannot be directly used to track feeble vibrations such as structural vibrations. This article proposes an effective increase in the sensitivity of the smartphone accelerometer utilizing the stochastic resonance (SR) phenomenon. SR is an approach where, counter-intuitively, feeble signals are amplified rather than overwhelmed by the addition of noise. This study introduces the 2D-frequency independent underdamped pinning stochastic resonance (2D-FI-UPSR) technique, which is a customized SR filter that enables identifying the frequencies of weak signals. To validate the feasibility of the proposed SR filter, an iPhone device is used to collect bridge acceleration data during normal traffic operation and the proposed 2D-FI-UPSR filter is used to process these data. The first four fundamental bridge frequencies are successfully identified from the iPhone data. In parallel to the iPhone, a highly sensitive wireless sensing network consists of 15 accelerometers (Silicon Designs accelerometers SDI-2012) is installed to validate the accuracy of the extracted frequencies. The measurement fidelity of the iPhone device is shown to be consistent with the wireless sensing network data with approximately 1% error in the first three bridge frequencies and 3% error in the fourth frequency.

## 1. Introduction 

Smartphone technology has advanced rapidly over the last decade. These ubiquitous devices are equipped with various sensors, ranging from accelerometers to magnetic field detectors. As a result, some researchers have investigated the viability of using smartphones data for various crowdsourcing remote sensing applications. One of the preliminary studies in this field is that done by Clayton et al. [1] and Kohler et al. [2]. They propose the “Community Seismic Network”, which is a community-based sense and response system for seismic activities using a distributed network of sensors in volunteers’ desktop computers, laptops and smartphones. The project aims to develop an early warning system for seismic events utilizing inexpensive sensors in volunteers’ devices, rather than using a small number of high-quality sensors. By processing the feedback from a huge number of “Seismic Network” sensors, they successfully characterize earthquake-induced vibrations and generate a ShakeMap (a map highlighting earthquake intensities across a city). In another application, Yu et al. [3,4] introduce the idea of structural health monitoring using smartphones. They use a smartphone as a mini-SHM system to collect information about the structure using iPhone sensors (accelerometers, inclinometers, temperature sensors, etc.) as well as using external sensors connected wirelessly to the iPhone. The authors use the iPhone data as an input to an algorithm embedded in the smartphone to perform “small-scale structure monitoring.” If smartphone readings cannot meet the monitoring criteria (e.g., the signal is corrupted by severe noise), the external sensor is used to collect high quality data from the structure. In a similar vein, Feng et al. [5] introduce the “Citizen Sensor*”* network, which is basically the use of smartphones as deployable accelerometers to collect structural vibrations. The authors use three commercial smartphones in versatile vibration tests. Their work proves the feasibility of using smartphones for the detection of moderate to large intensity vibrations. They succeed in identifying the first fundamental frequency for a full-scale bridge. 

While Yu et al. [3,4] and Feng et al. [5] put forward the concept of using smartphone accelerometers for SHM applications in their seminal papers, these research groups could only extract information about the first fundamental bridge frequency. For SHM applications information about other modes of vibration (second, third, fourth, etc.) is extremely useful in identifying structural damage. Lee et al. [6] show that by utilizing the first four frequencies for a cantilever beam, the location and the size of a crack can be precisely identified. Similarly, Nikolakopoulos et al. [7] identify structural damage in a single story frame from the shift in the first three fundamental frequencies. Furthermore, Yao et al. [8] proposed a power-free, passive wireless sensor that can be activated utilizing a radio frequency interrogator to track incipient damages in the structures. In addition, there are many other research works which conclude that using more than one mode of vibration can lead to great success in damage identification [9,10,11,12,13,14,15,16,17]. 

**Figure 1:**
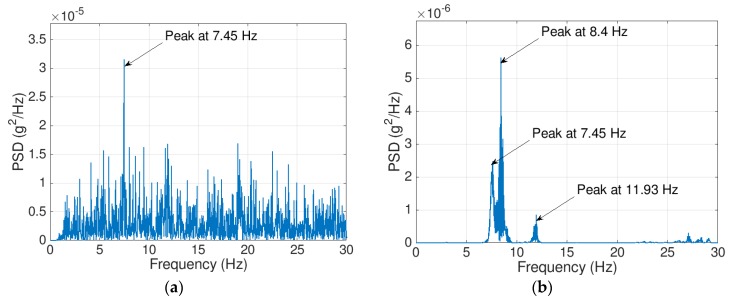
Power spectral density (PSD) of mid-span acceleration on a highway bridge recorded using (**a**) iPhone MPU-6500 and (**b**) SDI-2012.

There are two related issues with smartphone accelerometers that hinder their ability to detect several modes of vibration: a) low sensitivity and b) high output noise level. Figure 1 presents the power spectral density (PSD) plots for two acceleration signals for a highway bridge; the PSD of Figure 1a is for a signal recorded using an iPhone 6s, while that of Figure 1b is for a signal recorded using a Silicon Designs accelerometer (SDI Model 2012, manufactured by Silicon Designs Inc., Kirkland, WA, USA). The PSD for the iPhone signal has one clear peak at 7.45 Hz corresponding to the first fundamental bridge frequency, and no other peaks are evident at higher frequencies. In contrast, the SDI accelerometer shows three clear peaks corresponding to the first three fundamental frequencies of the bridge. This significant difference is due to the accelerometer sensitivity and the level of the output noise density. 

Accelerometer sensitivity is the ratio of change in sensor electrical output (signal) to mechanical input (e.g., acceleration), and it is measured differently in analog and digital accelerometers. An analog accelerometer produces a variable voltage related to the amount of acceleration applied to it. Digital accelerometers, on the other hand, perform an internal transformation to convert the voltage changes due to the applied acceleration directly into a digital format. Accordingly, an analog accelerometer’s sensitivity is measured in mV/g (milli-Volt per unit of acceleration), while a digital accelerometer’s sensitivity is measured in LSB/g (Least Significant Bit per unit of acceleration). The MPU-6500 in iPhones has a maximum sensitivity of 16,384 LSB/g. This is equivalent to 450 mV/g in an analog scale. The relation between the two measures is a function of the operating voltage and the scale of the analog to digital converter (ADC) where:

(1) $$\mathrm{LSB}=\frac{\mathrm{Reference}\;\mathrm{Voltage}}{2^{N\left(number\;of\;bits\right)}}$$

**Figure 2:**
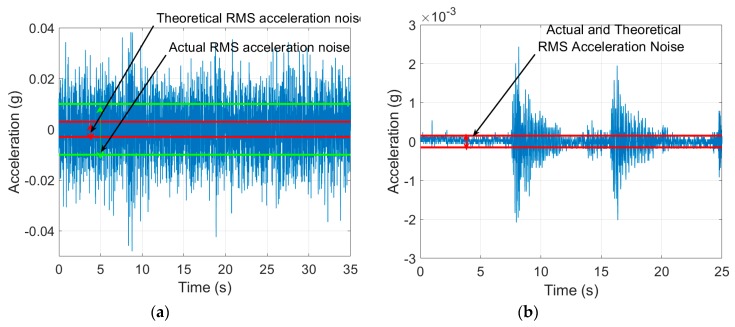
Mid-span acceleration of highway bridge recorded using (**a**) iPhone MPU-6500 and (**b**) SDI-2012. Strait lines show theoretical noise root mean square (RMS) value. Dashed lines show actual noise root mean square (RMS) value.

The MPU-6500 has an operating voltage of 1.8 V and the ADC scale is 16 bits. Thus, each unit of LSB is equivalent to 0.0275 mV and 16,384 LSB/g is equivalent to 450 mV/g. This is less than 25% of the sensitivity of the SDI-2012 which is 2000 mV/g. The difference in sensitivity is also evident in the acceleration plot (Figure 2). The amplitude of the acceleration measured using the SDI-2012 is as low as 10^−4^ g.

The iPhone accelerometer also has a high output noise density, as is evident in Figure 2. Output noise density equals the ratio of noise root mean square (RMS) to the square root of the scanning frequency:

(2) $$\mathrm{Noise}\;\mathrm{Density}=\frac{\mathrm{Noise}\;\mathrm{RMS}}{\sqrt{\mathrm{Scanning}\;\mathrm{Frequncy}\;\mathrm{Hz}}}\;\mu g/\sqrt{\mathrm{Hz}}$$

The MPU-6500 output noise density is 300 µg/$\sqrt{Hz}$ so, for a scanning frequency of 100 Hz, the noise RMS equals 3 × 10^−3^ g. This level is shown in the iPhone acceleration record of Figure 2a (the red straight lines). However, the actual noise RMS was measured by the authors and it is higher than that provided by the manufacturer, namely, 10^−2^ g (the dashed green lines in the figure). On the other hand, the SDI-2012 output noise density is 10 µg/$\sqrt{Hz}$. Therefore, for the same scanning frequency, the noise RMS is 10^−4^ g, as presented in Figure 2b (red straight lines). These two issues of sensitivity and noise limit the iPhone accelerometer’s capacity to extract frequencies for the bridge modes of vibration. 

This article proposes the exploitation of stochastic resonance to enhance the sensitivity of a smartphone’s accelerometer, which is seen as the first step in their potential use for structural health monitoring applications. Stochastic resonance (SR) is a counter-intuitive phenomenon as it uses background noise to effectively amplify signals that would otherwise be too weak for some sensors to be detected. In other words, SR utilizes noise to enhance the performance of the sensor. The phenomenon has been introduced in a study to interpret the periodicity of the ice age Benzi et al. [18]. Later on, researchers found that this phenomenon exists in the sensory system of many living organisms, such as crayfish [19] and crickets [20]. They found that these arthropods can amplify the feeble vibrations produced by the predators when the predator swim/fly to attack. These arthropods utilize SR to monitor vibrations at specific frequencies (associated with the attack swimming/flying motions of the predator). By monitoring the amplitude of the signal at that particular frequency, the arthropod can predict whether the predator is approaching or departing. If the amplitude increases, it indicates an approach and vice versa [20,21]. This intriguing phenomenon is currently attracting a great deal of interest in several research fields. However, there are two issues with traditional SR approach: 1) SR was originally developed for bi-stable, overdamped systems [22,23,24,25,26,27,28,29]. Civil Engineering structures tend to be mono-stable (one mode of vibration dominates the signal) and underdamped. 2) The frequency of the weak signal must be known in advance to extract the signal from the noise. To enable using SR in civil structural health monitoring applications, the authors introduce in a previous publication the frequency-independent underdamped pinning stochastic resonance (FI-UPSR) method, which is suitable for mono-stable, underdamped systems [30] and it is a frequency independent SR filter. In that study, FI-UPSR is successfully used to identify the fundamental bridge frequency using indirect measurements collected from an accelerometer mounted on an inspection vehicle passing over the bridge. 

In this article, a new version of the FI-UPSR is introduced to extract higher modes of vibrations from a signal recorded by an iPhone device. The new FI-UPSR, namely 2D-FI-UPSR, method does not require high computational power comparing to the original FI-UPSR method, and; therefore, it can be embedded to a smartphone application. This advantage will make SHM activities popular, since smartphones are more convenient to operate by the public. 

The manuscript commences by introducing the 2D-FI-UPSR concept. The 2D-FI-UPSR is then used to identify the frequencies of three weak signals. Finally, the 2D-FI-UPSR filter is used to process the iPhone acceleration where the first four frequencies for a full-scale concrete bridge are successfully identified.

## 2. Frequency Independent Underdamped Pinning Stochastic Resonance (FI-UPSR)

Stochastic resonance (SR) is a single degree of freedom filter that amplifies weak signals by resonating the noise with the signal. The general formula for stochastic resonance filter is:

(3) $$\frac{d^{2}x}{dt^{2}}=-V^{\prime }\left(x\right)-\gamma \frac{dx}{dt}+\left\{s\left(t\right)+n\left(t\right)\right\}$$

where x(t) is the extracted signal, γ is a damping term, s(t) is the original weak signal, and n(t) is the noise. Therefore {s(t)+n(t)} is the input (signal mixed with noise) to the stochastic resonance filter. The weak signal x(t) is extracted when it resonates with the input signal, {s(t)+n(t)}. This can be achieved either by tuning the input noise, or by changing the potential shape *V(x),* which can be done by tuning the force of the potential field (which is represented in Equation (3) by $V^{\prime }$(*x*)). Since the input noise cannot be controlled, the weak signal is extracted by changing the force of the potential field ($V^{\prime }$(*x*)). The force of the potential field is changed by adjusting the potential parameters, V*_d_*, *L*, and *x*_0_ that characterize the potential field [30,31]:

(4) $$V\left(x\right)=V_{0}-V_{d}\left(\exp\left(-\frac{{\left(x+x_{0}\right)}^{2}}{L^{2}}\right)+\exp\left(-\frac{{\left(x-x_{0}\right)}^{2}}{L^{2}}\right)\right)$$

**Figure 3:**
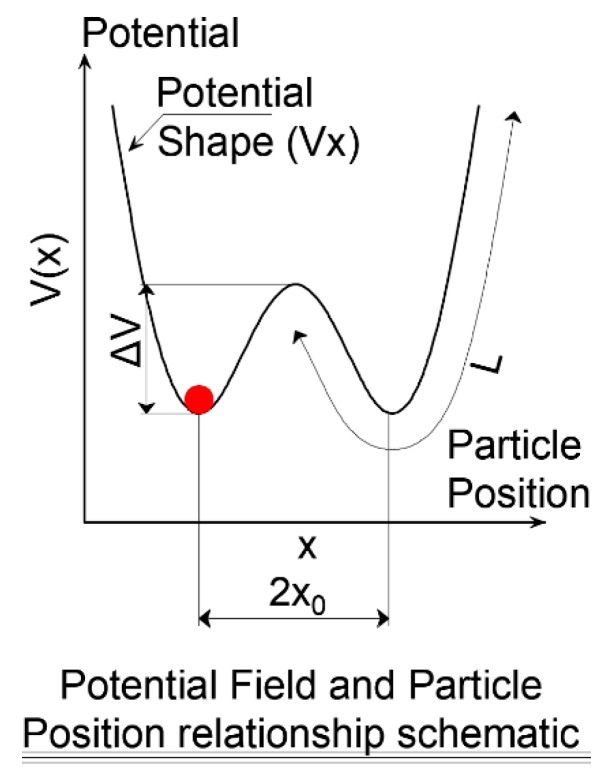
Potential shape for the underdamped pinning stochastic resonance for bi-stable condition.

**Figure 4:**
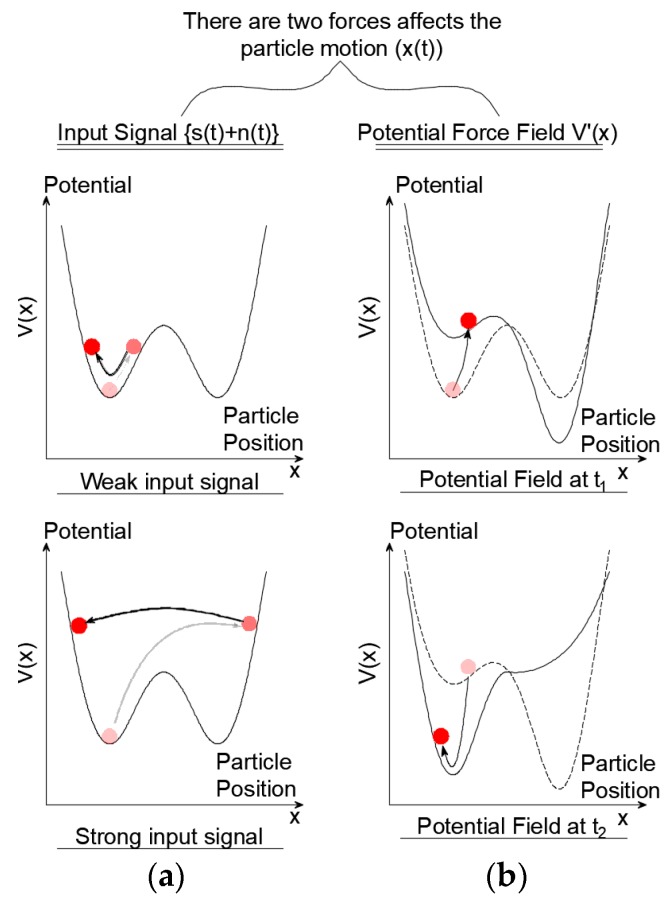
Potential shape for the underdamped pinning stochastic resonance for bi-stable condition (**a**) particle movement due to the input signal {*s*(*t*) + *n*(*t*)} (**b**) particle movement due to the force of the potential field $V^{\prime }$(*x*).

Figure 3 illustrates the terms of Equation (3). The position of the red particle represents the extracted signal, x(t), *L* is the length of one potential trough, *x*_0_ is the center of each pinning. The potential depth, *V_d_*, is an essential parameter for the filtering process. Higher *V_d_* values provide higher potential depths, in which the particle will not be able to surmount the barrier unless resonance is approached in Equation (3). The particle position is affected by two input forces; a) the input signal {s(t)+n(t)}, b) the force of the potential field $V^{\prime }$*(x)*. Figure 4 illustrates schematically the effect of these two forces on the particle. In Figure 4a the input signal {s(t)+n(t)} moves the particle along the potential shape. If the input signal is weak, the particle moves smoothly along the potential field. On the other hand, if the input signal is strong the particle will strongly hop from one position to another. Alternatively, the force of the potential field ($V^{\prime }$*(x)*) changes the shape of the field by moving the pinning points of the potential shape up and down. Figure 4b presents two shapes for the potential field at two-time steps. 

**Figure 5:**
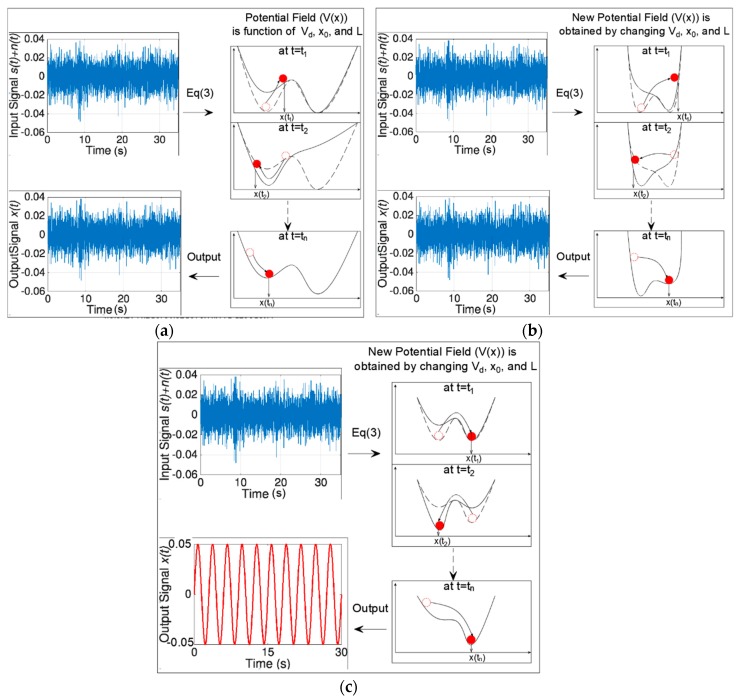
Extraction of the weak signal by adjusting the potential shape: (**a**) The potential shape has deep-wide troughs that prevents the particle from surmounting the potential barrier; (**b**) the potential shape has shallow-narrow troughs that allows the particle to hope on the potential wall; (**c**) the potential shape is adjusted where the particle motion is synchronized with the input force (particle motion is the extracted signal *x*(*t*), input force is the input signal *s*(*t*) + *n*(*t*)).

As previously noted, we cannot control the input signal {s(t)+n(t)}; therefore, the weak signal is extracted by changing the potential field (*V*(*x*)). Figure 5 presents three scenarios for three different potential fields, where the potential fields are generated by changing the potential parameters, V*_d_*, *L*, and *x*_0_. If the potential shape has a deep and wide trough, the particle will not be able to surmount the barrier and move from one potential minimum to another (Figure 5a), thus, resonance will not be approached. If the potential shape has a shallow and narrow trough, the particle will strongly hop on the potential shape as presented in Figure 5b. If the potential shape is properly adjusted, the input signal (or the input force) will synchronize with the particle’s hopping motion on the pinning pints of the potential shape. At this point, resonance is approached between the input signal and the extracted signal, as shown in Figure 5c. The following reference provides a graphical demonstration in real time for Figure 5c [32]. More information about the method can be found in the following references [30,31].

**Figure 6:**
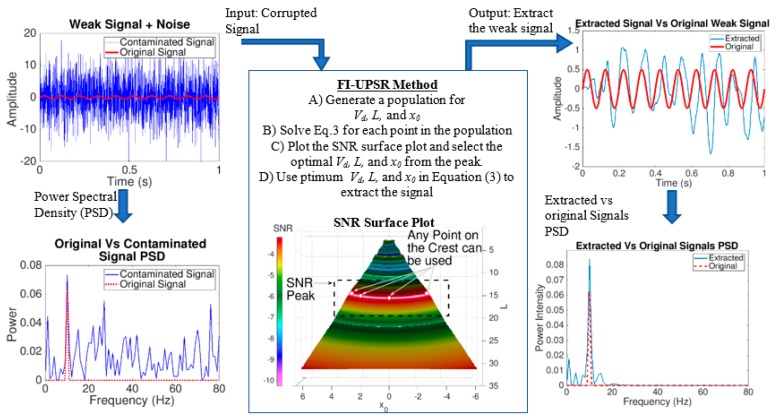
Frequency Independent Underdamped Pinning Stochastic Resonance (FI-UPSR) method for extracting feeble signals from strongly contaminated signal.

The weak signal is extracted by generating a set of potential shapes using different potential parameters (V*_d_*, *L*, and *x*_0_). Then, for each shape, the signal is extracted by applying Equation (3). Based on the previous publication [30], the authors recommend a range for the parameters of Equation (3) as presented in Table 1. These values will provide a proper potential shape that enables extracting the structure modes of vibrations. If the values are not properly selected, resonance will not be achieved in Equation (3) and thus the extracted signal will identically match the input signal as presented in Figure 5. The quality of the extracted signal is measured using the signal to noise ratio (SNR). The signal that has the highest SNR value, correspondingly has the smallest background noise. Therefore, the optimal potential parameters are those that maximize the SNR. In traditional SR methods, the SNR is calculated assuming that the target frequency of the weak signal is known. With FI-UPSR, feeble signals can be processed without knowing the target frequencies in advance. This is done using three dimensional surface plots of SNR. The SNR surface plot is created by a) generating a large population of the potential parameters (V*_d_* has a constant value, while *L* and *x*_0_ are varying), b) extracting the corresponding signal using Equation (3), and c) calculating the SNR values for the extracted signals. Having calculated the SNR for each point in the population (i.e., for point *i* with coordinates, (*x*_0_(*i*), *L*(*i*))), a three-dimensional surface plot is generated, and maximum values identified—see Figure 6. Any potential parameters on this peak can be utilized to extract the feeble signal. More information about the FI-UPSR method is available in reference [30]. Figure 6 presents an example for applying of FI-UPSR to extract a feeble signal. 

## 3. 2D-FI-UPSR Method

The FI-USPR framework needs high computational capabilities and it requires long processing time. For example, the time required to generate the SNR plot in Figure 6 is 491 s using a desktop computer. In order to embed this filter into a smartphone application that can be run directly by a smartphone processor, the FI-UPSR framework needs to be simplified. This section introduces the 2D-FI-UPSR method which is a two-dimensional SNR plot that can extract feeble signals and identify their frequencies. As illustrated in Figure 6, the SNR surface plot is radially symmetric about the origin. Thus, the 3D SNR plot can be simplified into a 2D SNR plot by eliminating the *x*_0_ coordinate. Further, it is possible to derive a direct relationship between the potential parameter, *L* and the frequency of the extracted signal. 

The derivative of the potential function in Equation (3) is:

(5) $$V^{\prime }\left(x\right)=\frac{dV\left(x\right)}{dx}=V_{d}\left(\frac{2\left(x+x_{0}\right)}{L^{2}}\exp\left(-\frac{{\left(x+x_{0}\right)}^{2}}{L^{2}}\right)+\frac{2\left(x-x_{0}\right)}{L^{2}}\exp\left(-\frac{\left(x-x_{0}\right)}{L^{2}}\right)\right)$$

Since the shape is symmetric around *x*_0_ = 0, it is possible to substitute, *x*_0_ = 0 in Equation (5), which yields:

(6) $$V^{\prime }\left(x\right)=\frac{4V_{d}}{L^{2}}x\exp\left(-\frac{x^{2}}{L^{2}}\right)$$

For a relatively large *L*, the exponential term approaches zero and Equation (6) can be approximated as:

(7) $$V^{\prime }\left(x\right)=\frac{4V_{d}}{L^{2}}x$$

Substituting Equation (7) into Equation (3), and rearranging gives:

(8) $$\frac{d^{2}x}{dt^{2}}+\gamma \frac{dx}{dt}+\frac{4V_{d}}{L^{2}}x=\left\{s\left(t\right)+n\left(t\right)\right\}$$

Equation (8) is in the form of forced damped vibration for a single degree of freedom system, where the system will resonate when the system frequency equals that of the applied force (in this case, s(t)+n(t)). Therefore, the frequency of the extracted signal, *x*(*t*) equals the natural frequency for a single degree of freedom system:

(9) $$\omega =2\pi f=\sqrt{\frac{4V_{d}}{L^{2}}}$$

Therefore:

(10) $$f=\sqrt{\frac{V_{d}}{\pi^{2}L^{2}}}$$

**Figure 7:**
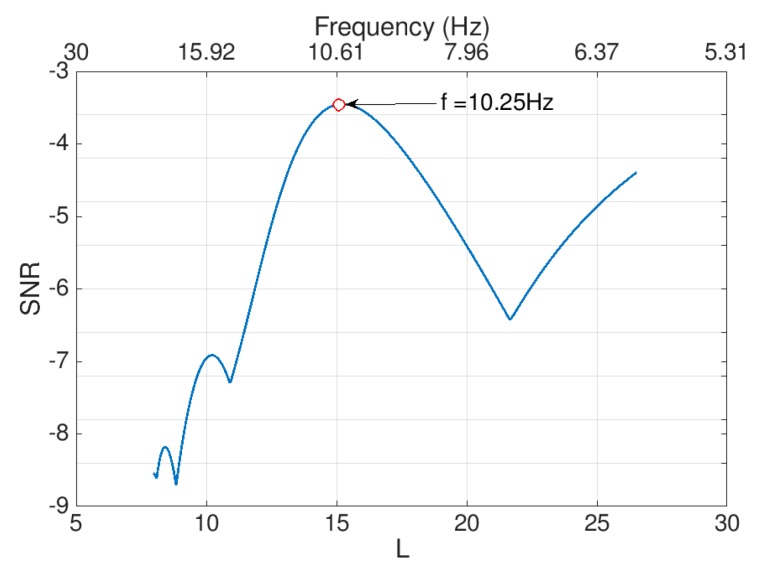
2D SNR plot produced using the 2D-FI-UPSR method.

Equation (10) provides a direct relationship between the potential parameter *L* and the frequency of the extracted signal. By eliminating *x*_0_ from the graph and introducing this relationship, the SNR surface plot of Figure 6 can be simplified to that of Figure 7. Along with the simplicity of Figure 7, the processing time drops from 490 to 1.1 s.

In summary, a simpler 2D SNR plot is derived by setting *x*_0_ = 0 and by directly calculating the resonant frequency of the extracted signals. The following points summarize the steps of reproducing the 2D SNR plots:

(1) Select values for γ, and *V_d_* (recommended ranges are 0.1 > γ > 0.7, *V_d_* > 100)
(2) Identify the limits for the scanning window (*f_min_* and *f_max_*)
(3) Using Equation (10), find the corresponding values for the potential parameter, *L* (*L_min_* and *L_max_*)
(4) Generate a population for *L* between the two boundaries
(5) Solve Equation (3) for each point in *L(i)* in the population (a numerical discretization for Equation (3) is available in [30,31])
(6) Calculate the SNR function for the extracted signal *x*(*t*)
(7) Plot SNR versus *L* and the frequency to generate Figure 7

As shown in this figure, the SNR peaks correspond directly to the frequency of the feeble signal. This approach will be followed in this paper to extract the bridge frequencies from the iPhone accelerations.

## 4. Applying the 2D-FI-UPSR to Identify the Frequencies of Multiple Feeble Signals

**Figure 8:**
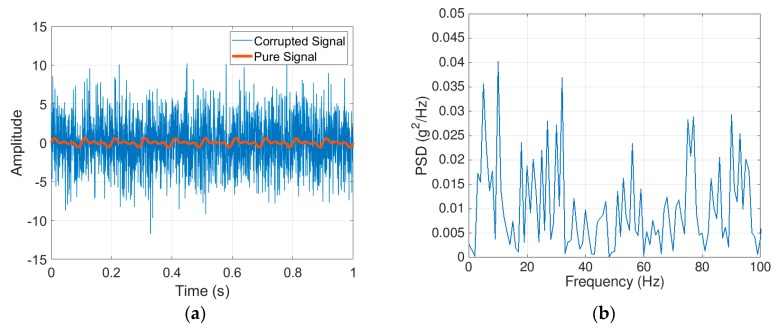
Weak signal corrupted by severe background noise: (**a**) Input signal, (**b**) power spectral density for the corrupted signal.

Previous research in the stochastic resonance field focuses on the detection of a single frequency in one weak signal. This section investigates the utilization of 2D-FI-UPSR for the detection of multiple frequencies of feeble signals. Three sinusoidal functions represent the feeble signals. The frequencies and amplitudes are listed in Table 2. The signals are combined and then corrupted with a −10 dB Gaussian white noise. Figure 8a presents the pure combined signal and the corrupted signal. The power spectral density (PSD) in Figure 8b is raucous and the frequencies of the weak signals cannot be identified.

**Figure 9:**
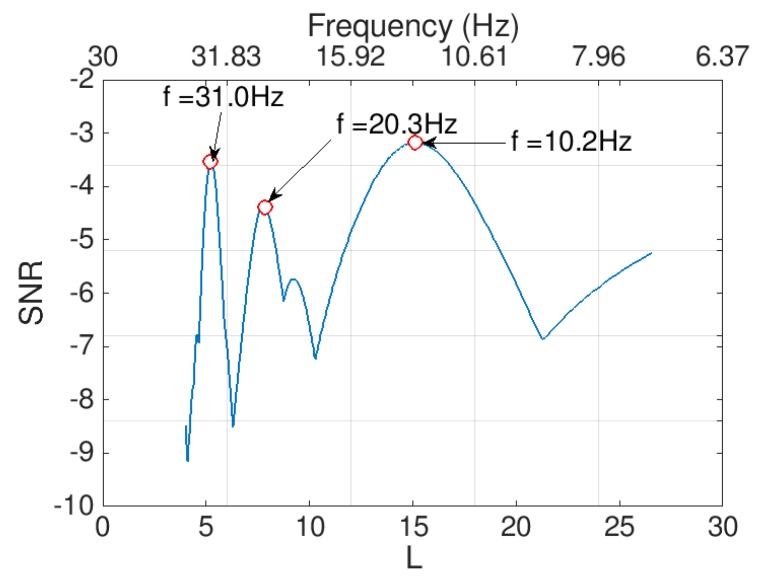
2D-FI-UPSR plot for signal mixed with extreme noise, Signal to Noise Ratio (SNR) in the y axis, L potential parameter and the associated signal frequencies in the x axis.

To extract the frequencies from the feeble signal utilizing the 2D-FI-UPSR method, the following parameters are used as an input to the algorithm: *γ* = 0.5, *V_d_* = 100, and the frequency window is varied from 5 to 30 Hz. As previously mentioned, a population for the *L* parameter is generated, then Equation (3) is solved repeatedly for each point in the population to extract the corresponding signal. Afterwards, the SNR value for each extracted signal is calculated to plot the 2D SNR plot. Feeble signals can provide a clear peak in the 2D SNR plot, as can be seen in Figure 9. Three peaks are evident at the feeble signal frequencies (i.e., 10, 20, and 30 Hz). It can be seen that the approach has succeeded in identifying all three frequencies. In the following section the 2D-FI-UPSR approach will be utilized to extract the fundamental bridge frequencies from acceleration data recorded directly by an iPhone device.

## 5. Investigating the Feasibility of Using the 2D-FI-UPSR in Identifying the Fundamental Bridge Frequencies Using Acceleration Data Recorded by an iPhone Device

**Figure 10:**
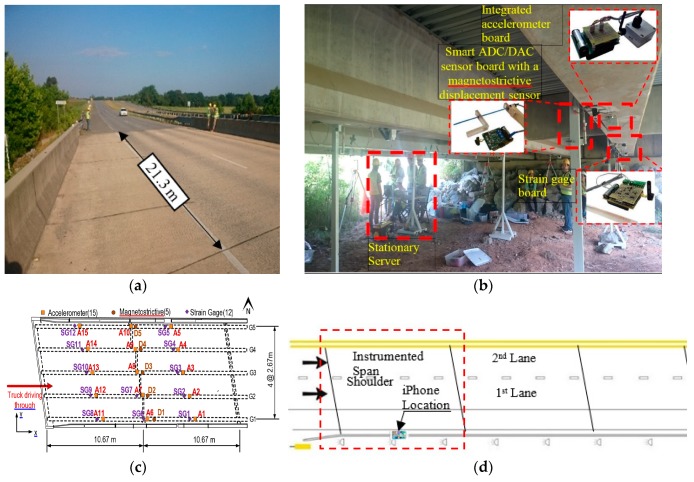
Field instrumentation: (**a**) Bridge overview, (**b**) instrumentation, (**c**) plan view of instrumentation on the girders, (**d**) iPhone location.

A field test was carried out on a skewed prestressed concrete bridge consisting of three simply supported spans. The bridge is located on HWY 113 in Bartow County, Atlanta, Georgia between Covered Bridge Rd and Dry Creek Rd. Each span of the bridge is 21.3 m, center to center of the supports. An in-situ reinforced concrete slab connects the five prestressed concrete girders in each case. Each carriageway consists of two lanes of one-way traffic and one hard shoulder. The instrumentation was installed on the first span from the traffic direction, as shown in Figure 10. Each girder was equipped with three Silicon Designs accelerometers (Model: SDI-2012), spaced equally along the length of the girder. The accelerometers were connected to a wireless sensor board to transmit the data to the data acquisition station which lies underneath the bridge [33,34].

**Figure 11:**
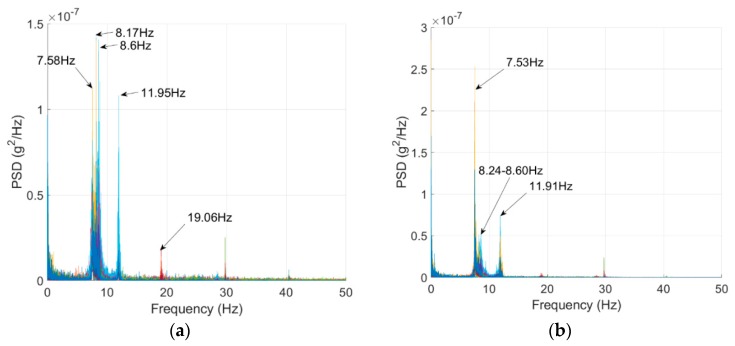
Power spectral density of the bridge vibration signals: (**a**) Test 1, (**b**) Test 2.

**Figure 12:**
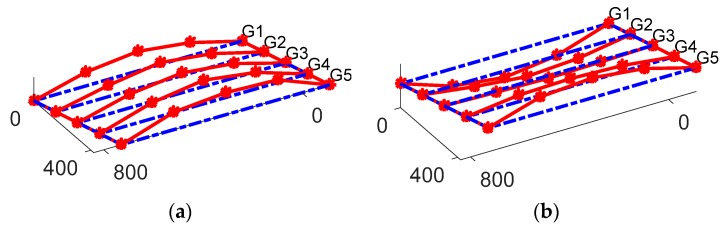

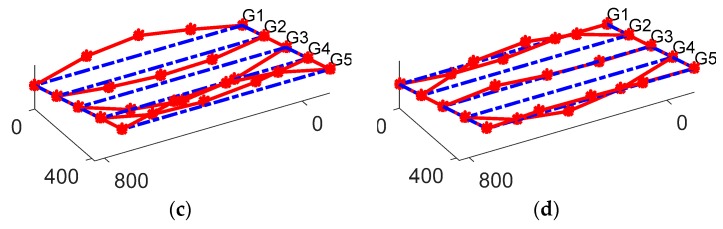
Natural frequencies and mode shapes of the bridge corresponding to frequencies of (**a**) 7.56 Hz, (**b**) 8.4 Hz, (**c**) 11.93 Hz, (**d**) 19.04 Hz [33,34].

**Figure 13:**
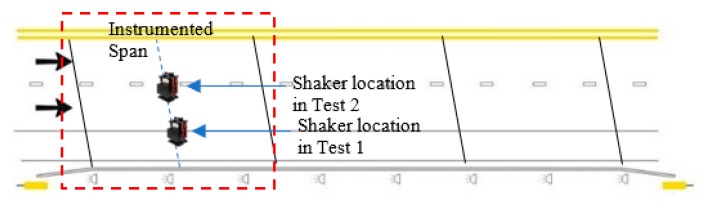
Shaker location in the two tests.

The bridge frequencies were identified using a vibration test. An ELECTRO-SEIS Long Stroke Exciter vibration shaker was utilized to excite the bridge, while the bridge accelerations were recorded. The scanning frequency for the SDI-2012 accelerometers was set to 100 Hz; the frequency resolution was 6.25 × 10^−3^ Hz_._ The traffic was blocked during the test as recommended by Yang et al. [35] and the entire process repeated twice. The PSD’s of the SDI-2012 raw signals are illustrated in Figure 11. The 2D-FI-UPSR is not applied to this signal since the SDI-2012 accelerometer has very high sensitivity and low output noise density. The bridge frequencies are listed in Table 3 and the mode shapes are presented in Figure 12. There are four modes of vibration for the bridge; two longitudinal modes (at 7.56 and 11.93 Hz), and two transverse modes (at 8.4 and 19.04 Hz). At Test 1, the shaker was close to the bridge shoulder; therefore, the longitudinal and the frequencies corresponding to the transverse modes of vibration are observed in the PSD. On the other hand, in Test 2, the shaker was placed above G3 girder (see Figure 13). At this location the shaker induces more energy for the longitudinal modes; therefore, the peak at 8.4 Hz is dramatically reduced while no peak is observed at 19.04 Hz. Since the smartphone is placed on the bridge barrier, it is expected that traffic configuration will have the same effect on the iPhone data. This point will be revisited in this article after presenting the frequencies obtained by the iPhone device. 

**Figure 14:**
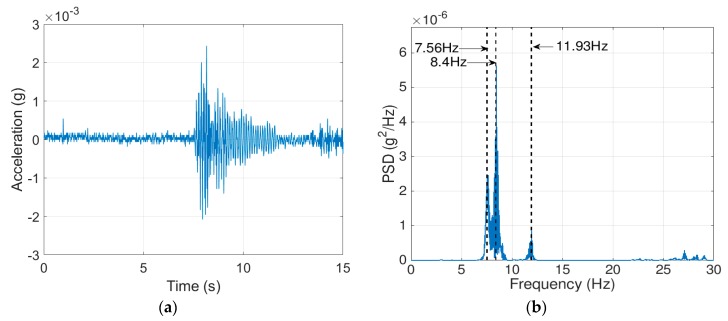
Acceleration recorded using the SDI-2012: (**a**) Acceleration, (**b**) corresponding Power Spectral Density (PSD).

**Figure 15:**
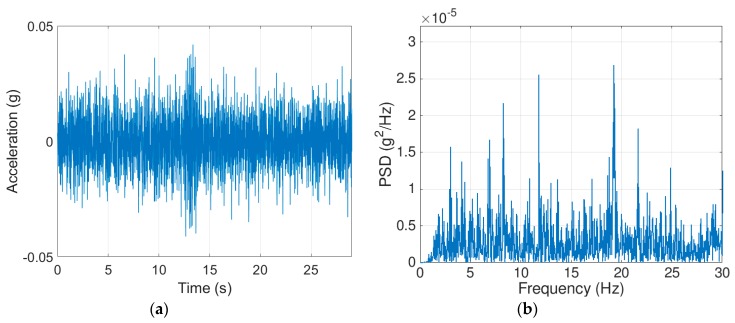
Acceleration recorded using the iPhone device: (**a**) Acceleration, (**b**) corresponding PSD.

To investigate the fidelity of the proposed 2D-FI-UPSR method, an iPhone 6s was used to record the accelerations while normal traffic was travelling over the bridge. As previously mentioned, the iPhones 6s is equipped with the MPU-6500 MEMS digital accelerometer. The iPhone acceleration was recorded using the VibSensor® program which was downloaded from the App Store®. Other software, Seismometer® was used to extract comparable data. The iPhone was attached to the bridge rail using a double-sided adhesive sheet. In order to assess the quality of the iPhone data, the SDI-2012 (SG6 in Figure 10c) was used to record the bridge vibration under the traffic load, simultaneously with the iPhone device. This accelerometer (SG6) is chosen because it is the closest accelerometer to the iPhone location (except that the accelerometer was placed on the bottom of girder No. G5, while the iPhone was on top of the bridge rail). Figure 14 and Figure 15 present a sample of data for the recorded vertical accelerations by the SG6 and the iPhone, respectively. The PSD for the SG6 accelerometer shows clear peaks at the fundamental bridge frequencies. However, the PSD for the iPhone signal (Figure 15) is noisy and shows several peaks, making it difficult to identify the bridge frequencies. 

To measure the quality of the iPhone acceleration, we use the SDI-2012 signal as a reference to calculate the SNR for the iPhone signal using the following equation:

(11) $$SNR=10\log_{10}\frac{\sum_{0}^{N/2}A_{x}}{\sum_{0}^{N/2}A_{s+n}}$$

where $\sum_{0}^{N/2}A_{x}$ is the sum of signal power, while $\sum_{0}^{N/2}A_{s+n}$ is the sum of the noise power. Using Equation (11), the SNR for the iPhone signal is approximately –80 dB. Furthermore, the SDI-2012 has much higher sensitivity as can be seen in the figures—the magnitude of the bridge acceleration is varying in the range ±0.003 g for the SDI-2012, while the iPhone acceleration record fluctuates in the range ±0.03 g. This proves that a pure iPhone signal cannot be used directly in identifying the bridge frequencies. 

**Figure 16:**
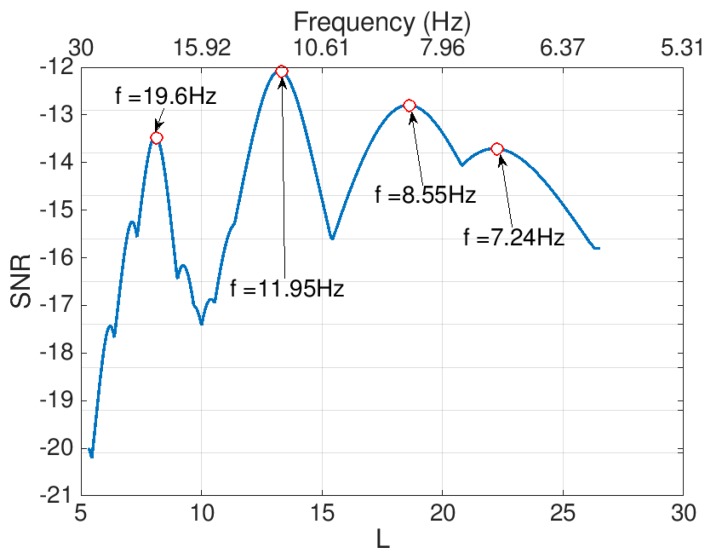
Two-dimensional plot of Signal to Noise Ratio (SNR) (Test 1).

**Figure 17:**
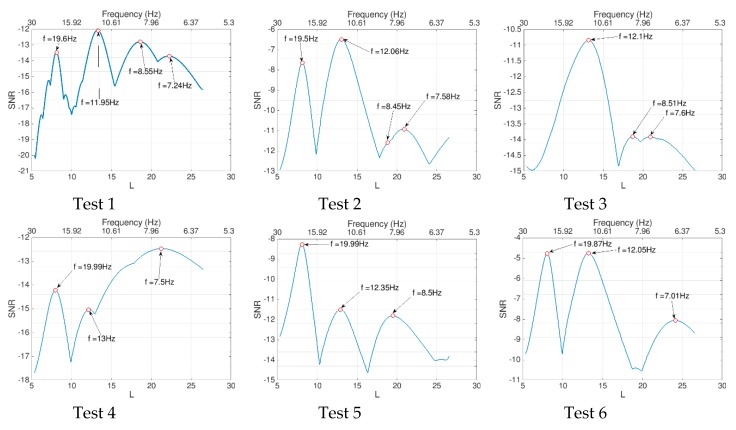

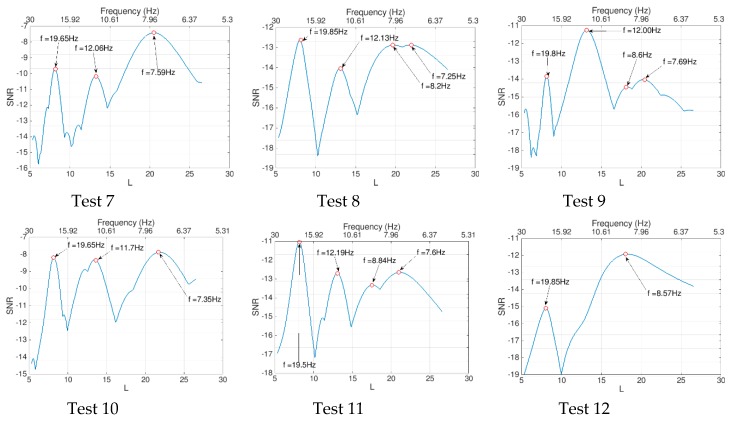
2D-FI-UPSR plots and corresponding extracted frequencies.

In order to extract the bridge frequencies using the iPhone, the signal of Figure 15 is processed using the proposed 2D-FI-UPSR method. The 2D SNR plot is produced using the steps described in Section 3 and is presented in Figure 16. As can be seen, there are four peaks in the SNR plot, corresponding to the first four fundamental bridge frequencies. By comparing Figure 16 with the PSD in Figure 15b, the 2D-FI-UPSR method shows great success in extracting the fundamental bridge frequencies from the iPhone acceleration. The iPhone was used to record the bridge acceleration for twelve crossings of different vehicles over the bridge. The iPhone location was not changed during the test. The collected accelerations were processed using the proposed 2D-FI-UPSR method to extract the fundamental bridge frequencies. Figure 17 presents the 2D SNR plots for the twelve crossings and Table 4 presents the corresponding traffic configurations. As Figure 17 presents, the four fundamental frequencies are not identified in all tests. The first four fundamental frequencies are identified in Tests 1, 2, 8, 9, and 11. This is due to the presence of heavy multiple vehicles that vibrate all four modes of vibrations. However, in Tests 2 and 8 it is quite challenging to identify the peaks of the two first frequencies (the 7.54 Hz and the 8.4 Hz). On the frequency scale, these two frequencies are too close to each other; therefore, it is challenging to distinguish between them in the 2D SNR plots. This is evident in the SNR plots, where the trough between the first two frequencies is not as clear as the troughs between the remaining frequencies. Furthermore, in Tests 4, 6, 7, and 10 the second frequencies are not identified in the 2D SNR plot. This can be ascribed to the traffic configuration, the iPhone location and the orientation of these particular modes of vibration. The first frequency (at 7.54 Hz), as presented in Figure 12a, is associated with the first longitudinal mode of vibration, while the second frequency (at 8.4 Hz) is associated with the first transverse mode of vibration. Additionally, as presented in Figure 10, the iPhone is located laterally in the center and to the right side of the traffic. When the traffic passes through “lane 1” (see Figure 10d), the iPhone is influenced more by longitudinal modes of vibration. On the other hand, when the traffic passes through “lane 2”, the iPhone is influenced more by transverse modes of vibration. This effect was also observed during the free vibration test as previously discussed in this paper. By viewing the traffic configuration in Table 4 for Tests 4, 6, 7, and 10, it can be seen that the traffic is passing through “lane 1.” Therefore, the first bridge frequency is identified in the 2D SNR plot, while the second bridge frequency is not identified. The opposite is true for Tests 5 and 12. To overcome this issue, it is recommended to take readings from multiple locations on the bridge (e.g., from the right and the left rails, also from the mid-point and the quarter points along the span). 

**Figure 18:**
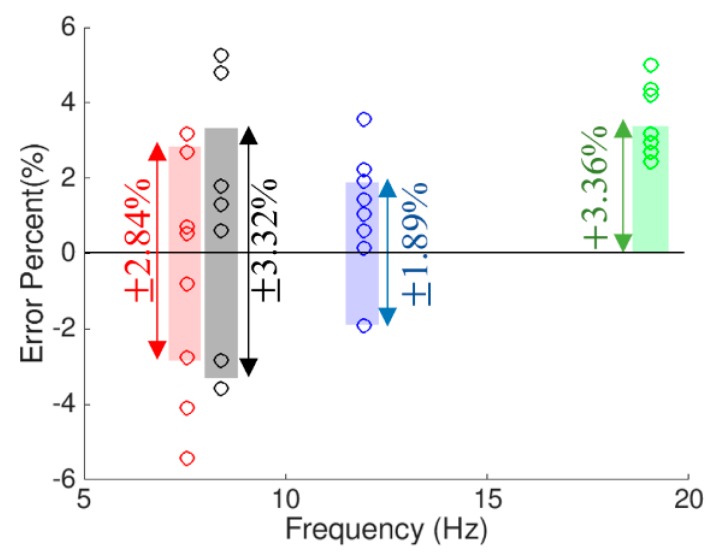
Error percentage in identifying the bridge frequency.

**Figure 19:**
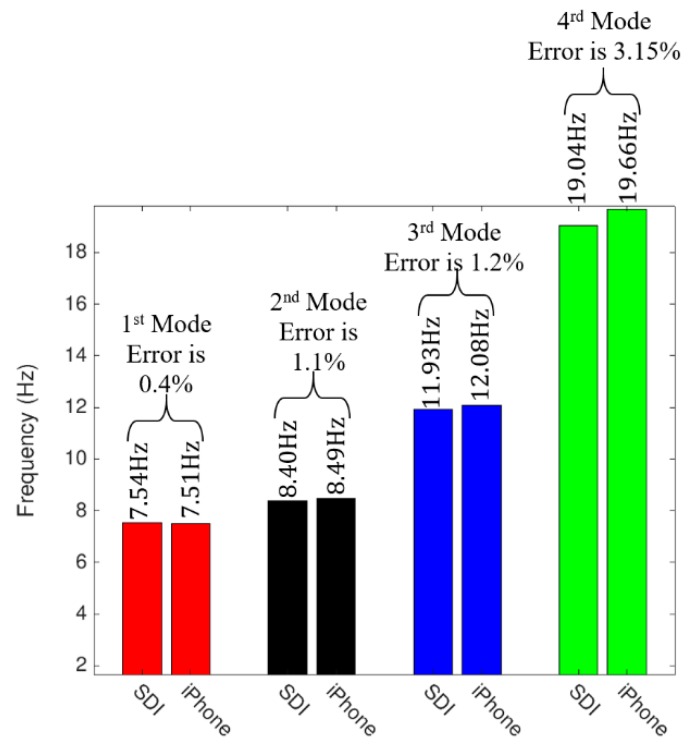
Comparison of the mean bridge frequencies extracted by the Silicon Designs and the iPhone devices.

To measure the accuracy of the iPhone device after applying the 2D-FI-UPSR method, the extracted frequencies for each mode of vibration are grouped into four clusters. The accuracy in identifying the frequency is measured separately for each cluster. The error percentage is calculated with respect to the frequencies obtained by the free vibration test using the SDI-2012 accelerometers. The error percentage in the calculated frequencies was evaluated after eliminating the outliers and is presented in Figure 18. The shaded areas present the 67% confidence interval for the error in the extracted frequencies. The third mode has the lowest error percentage (the 67% confidence interval boundaries are ±1.89%), while the fourth mode has the highest error percentage (the 67% confidence interval boundary is +3.36%). The mean frequency for each cluster is compared to the mean bridge frequencies that were extracted by the SDI accelerometer and presented in Figure 19. The error percentage between the two devices for the first frequency is only 0.4%. For the second and the third frequencies, the error percentage between the two devices is around 1%. However, the fourth frequency has a relatively high error compared to the first three fundamental frequencies (i.e., 3.15%). Regardless of the latter point, the results show that utilizing the 2D-FI-UPSR method in processing the iPhone signal significantly improves the device sensitivity and enables the extraction of more than one fundamental frequency for the bridge with acceptable accuracy. It is worth mentioning that the smartphone can provide environmental data during the test. Monitoring the environmental condition could enhance the detection of structural degradation by providing data with which to filter out these effects. Previous research shows that, following this approach, structural degradation can be precisely detected by monitoring the bridge frequencies [36]. 

## 6. Conclusions

This article investigates the feasibility of utilizing smartphones for SHM applications. More specifically, it investigates the potential for a low-grade iPhone accelerometer to extract the first four fundamental frequencies of a highway bridge. Since the iPhone MEMS accelerometer has a very low sensitivity as well as a relatively high output noise level, the raw acceleration could not be used directly to extract the bridge frequencies. In this paper, stochastic resonance is used to enhance the sensitivity of the iPhone signal by suppressing the associated noise. The authors introduce the 2D-FI-UPSR framework for the extraction of feeble signals using accelerations recorded directly by a smartphone device. The proposed framework is then used to identify the first four fundamental frequencies for a full-scale bridge using accelerations recorded by an iPhone device. The 2D-FI-UPSR framework was applied for twelve iPhone accelerations and succeeds in identifying the first four bridge frequencies. To evaluate the accuracy of the extracted frequencies, the authors used a wireless sensing network consisting of 15 high quality accelerometers to measure the exact frequencies of the bridge. The accelerometer (Model SDI-2012) has a much higher sensitivity (four times higher than the iPhone MPU-6500 accelerometer) and a much lower output noise density (1/30 times less than the iPhone MPU-6500 accelerometer). The difference between the bridge frequencies obtained by processing the iPhone accelerations using the 2D-FI-UPSR method, and those obtained using the SDI accelerometers is within the 1% margin. This small difference proves the potential of the proposed 2D-FI-UPSR framework to increase smartphone sensitivity. Furthermore, the proposed method is suitable for the smartphone’s limited processing capabilities. This feature promises to develop a smartphone application that can directly infer the bridge frequencies by attaching the smartphone to the bridge rails. The goal of this article is to pave the way for integrating smartphone technology into SHM applications.

## Figures and Tables

**Table 1:** Recommended initial values for *V_d_*, and γ.

*V_d_*	γ
≥100	0.1–0.7

**Table 2:** Test signal frequencies and amplitudes.

Signal	Amplitude	Frequency
First	0.25	10 Hz
Second	0.25	20 Hz
Third	0.25	30 Hz

**Table 3:** Average bridge frequency (Hz).

First	Second	Third	Fourth
7.56	8.4	11.93	19.04

**Table 4:** Extracted bridge frequencies (Hz).

Test	Vehicle Type	Vehicle on the Bridge	Extracted Bridge Frequency			
			1st	2nd	3rd	4th
1	2 P *	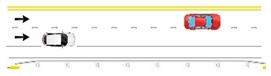	7.24	8.55	11.95	19.6
2	1 SUV **	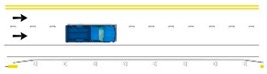	7.58	8.45	12.06	19.5
3	1 SUV	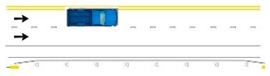	7.6	8.51	12.1	--
4	1 P	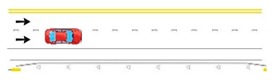	7.5	--	13	19.99
5	1 P + 1 SUV	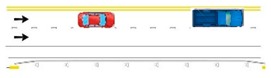	--	8.5	12.3	19.99
6	1 P + 1 SUV	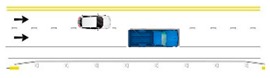	7.01	--	12.05	19.87
7	1 P	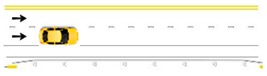	7.59	--	12.06	19.65
8	1 Truck + 1 SUV	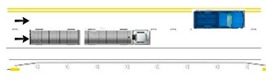	7.25	8.2	12.13	19.85
9	3 Ps	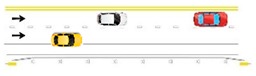	7.69	8.6	12.00	19.8
10	1 P	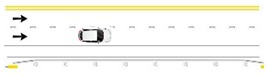	7.35	--	11.7	19.65
11	2P	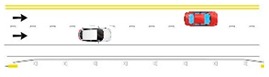	7.6	8.84	12. 19	19.5
12	2 Trucks	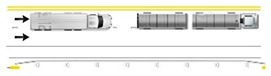	--	8.57	--	19.85

* P indicates a passenger vehicle; ** SUV indicates a sport utility vehicle
